# Impact of the COVID-19 pandemic on breast and cervical cancer stage at diagnosis in Brazil

**DOI:** 10.3332/ecancer.2021.1299

**Published:** 2021-10-04

**Authors:** Renata Colombo Bonadio, Ana Paula Messias, Otavio Augusto Moreira, Letícia Vecchi Leis, Bruna Zanin Orsi, Laura Testa, Maria Del Pilar Estevez-Diz

**Affiliations:** Medical Oncology - Instituto do Cancer do Estado de Sao Paulo (ICESP), Av. Dr. Arnaldo, 251, São Paulo – SP, 01246-000, Brazil; † These authors contributed equally to this work

**Keywords:** COVID-19, pandemic, breast cancer, cervical cancer, stage, diagnosis

## Abstract

**Background:**

The COVID-19 pandemic has led to the need for health services adjustments, which may have compromised management of other diseases. For cancer patients, delays may significantly impair outcomes in some situations. We aimed to assess the impact of the COVID-19 pandemic in breast and cervical cancer diagnosis and treatment compared to the same period prior to the pandemic.

**Methods:**

Data were collected from patients attending their first visit to a Brazilian cancer centre from 1 September 2020 to 31 January 2021 and from 1 September 2019 to 31 January 2020. The pandemic started in February 2020 in Brazil and is still ongoing. We considered this period (September/20–January/21) to be representative of the pandemic impact on cancer management. The primary endpoint was breast and cervical cancer stages at diagnosis.

**Results:**

A total of 268 breast cancer patients and 44 cervical cancer patients had their first consult in our cancer centre from September/20 to January/21; 457 and 60, respectively, occurred from September/19 to January/20. Patients who attended their first visit during the pandemic (September/20–January/21) presented with more advanced-stage breast cancer (*p* < 0.001) and cervical cancer (*p* = 0.328) than those in the period prior to the pandemic (September/19–January/20), although the difference was not statistically significant for cervical cancer. The proportion of cervical cancer patients diagnosed with locally advanced disease (stages III–IVA) was 56.8% (*N* = 25) in September/20–January/21 compared to 43.3% (*N* = 26) in September/19–January/20. Similarly, 37.3% (*N* = 100) of breast cancer patients had stage III disease in September/20–January/21 compared to 23.2% (*N* = 106) in September/19–January/20. Fewer breast cancer patients (13.7%) were diagnosed due to screening tests during the pandemic than before it (25.5%) (*p* < 0.001).

**Conclusions:**

Breast and cervical cancer patients had more advanced-stage diseases in their first visit to a cancer centre during the COVID-19 pandemic compared to a similar period prior to the pandemic. Efforts should be made not to compromise essential cancer services since this results in long-term negative impacts for oncologic patients.

## Introduction

Since the beginning of the COVID-19 pandemic, health services have taken measures to increase the admission capacity of COVID-19 patients and reduce non-COVID-19 patients’ circulation. Measures with this aim included reducing outpatient visits and postponing exams, procedures and elective surgeries [1]. Cancer patients can be particularly affected by these measures since the time to diagnosis and treatment initiation can negatively impact outcomes. National screening programmes were temporarily suspended in some places to decrease the health system demand [2]. The Brazilian National Health Agency (Agência Nacional de Saúde – ANS) recommended the postponement of visits, exams and procedures that were not urgent during quarantine [3]. In São Paulo state, the quarantine initiated on 17 March 2020 and ended on 18 August 2021 [4]. In addition, new cases investigation was also delayed by the reorganisation of health services and patients’ fear of contacting COVID-19.

A significant drop in the number of screening tests and, consequently, in the number of diagnoses of cancerous and precursor lesions in pandemic times in the United States has already been described [5]. In a conservative analysis of the effect of COVID-19 on the screening and treatment of breast and colorectal cancer in the United States, it is estimated that almost 10,000 deaths will occur in excess in the next 10 years, that is, an increase of approximately 1% in the number of deaths, during a period when we would expect to see almost 1,000,000 deaths from these two tumours [6].

Breast cancer and cervical cancer are two neoplasms with a high incidence, being the first and third most common neoplasms among women in Brazil in 2020 [7]. For both, effective screening strategies are available through mammography and oncotic colpocytology, respectively. In Brazil, breast cancer screening is recommended for women from 50 to 74 years with biannual mammography. For cervical cancer, screening with oncotic colpocytology is recommended for sexually active women from 25 to 64 years, with a frequency of every 3 years after two normal exams with 1 year interval. Both these screening exams are provided by the Brazilian public health system. In case of abnormal results, patients are submitted to a biopsy in a primary or secondary health service, and directed to a tertiary cancer centre in case of a positive result for malignancy. In addition, for cervical cancer, the human papillomavirus (HPV) vaccine is also an effective instrument of primary prevention. In many countries, HPV vaccination occurs in schools, which had their activities interrupted during the pandemic. Thus, with the reallocation of health resources during the COVID-19 pandemic, the interruption and delay in primary and secondary prevention procedures may have had a negative impact on the diagnosis of such patients.

We aimed to evaluate the impact of the COVID-19 pandemic in the stage at diagnosis and access to cancer services of new breast and cervical cancer cases.

## Methods

### Study design and participants

Patients who were diagnosed and attended their first consult at the Instituto do Câncer do Estado de São Paulo (ICESP – São Paulo, Brazil), from 1 September 2020 to 31 January 2021 and from 1 September 2019 to 31 January 2020, were evaluated in this cross-sectional study. ICESP is a tertiary cancer centre that assists over 10,000 new cancer patients yearly and has 499 hospitalisation beds. The service provides all exams, procedures and treatments (including surgery, radiotherapy and chemotherapy) related to cancer care.

The first case of severe acute respiratory syndrome coronavirus 2 infection in Brazil occurred in February 2020, and the COVID-19 pandemic is still ongoing in the country until the date of this manuscript elaboration. Therefore, the period from September 2020 to January 2021 was considered representative of the impact of the COVID-19 pandemic on cancer diagnosis. In this period, the pandemic had been present for at least 6 months in Brazil, leading to measures of social distancing and health care services readjustments to attend COVID-19 patients.

Patients included in the study had histologically confirmed breast or cervical carcinoma of any histologic type. Exclusion criteria were previous oncologic treatment in another service (except for upfront surgery for the primary tumour) and a second active primary malignancy diagnosis. Electronic records were reviewed for data collection.

The study objective was to evaluate if new breast and cervical cancer cases had more advanced stages at diagnosis in their first visit to a cancer centre during the COVID-19 pandemic. The primary endpoint was breast or cervical cancer stages in their first visit to a cancer centre from September 2020 to January 2021 compared to September 2019 to January 2020. Breast cancer staging was defined according to the American Joint Committee on Cancer and the Union for International Cancer Control (AJCC/UICC) 8th edition tumour, node, metastasis (TNM) anatomic Staging System. Cervical cancer staging was based on the 2018 International Federation of Gynecology and Obstetrics (FIGO) Staging System. The secondary endpoint was the interval from tumour biopsy to the first cancer centre visit.

The Local Ethics Committee approved the study.

### Statistical analysis

Patients’ data were summarised using descriptive statistics. Continuous variables are presented using median and range, while categorical variables are presented using absolute numbers and percentages.

Tumour stages between the two periods were compared using the Chi-squared test. Intervals from biopsy to first cancer centre visit were treated as continuous variables. Shapiro–Wilk test was used to evaluate its normality. Data with normal distribution were compared using the Student *t*-test. Mann–Whitney test was used for data with skewed distribution. Statistical analyses were performed with Stata software (StataCorp, Texas, USA), version 15.1. *p* values < 0.05 were considered statistically significant.

## Results

### Breast and cervical cancer stages

From September/20 to January/21, during the pandemic period, 268 breast cancer and 44 cervical cancer patients attended their first visit to the cancer centre. In comparison, 457 breast cancer and 60 cervical cancer patients had their first visit during the same period in the previous year (September/19–January/20).

**Figure 1.**
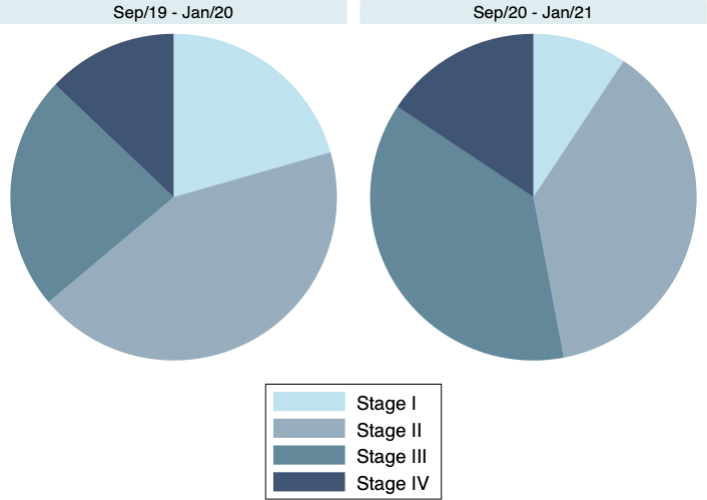
Breast cancer stages during (September/20–January/21) and prior to (September/19–January/20) the COVID-19 pandemic, according to the AJCC/UICC 8th edition TNM anatomic Staging System.

Breast cancer patients had more advanced tumour stages during the pandemic at the first visit compared to the same period prior to the pandemic (*p* < 0.001). For instance, the proportion of breast cancer patients with stage III disease was 37.3% (*N* = 100/268) in September/20–January/21 and 23.2% (*N* = 106/457) in September/19–January/20. Accordingly, only 9.3% (25/268) had stage I disease during the pandemic versus 20.6% (94/457) in September/19–January/20. The proportion of patients in each TNM category is presented in Table 1 and shown in Figure 1.

Breast cancer diagnosis was made after routine screening tests in 25.5% of the patients in September/19–January/20. This proportion was considerably lower in September/20–January/21, with 13.7% diagnosed after screening tests (*p* < 0.001). During the pandemic, most patients were diagnosed with breast cancer due to symptomatic presentation (86.3%).

**Figure 2.**
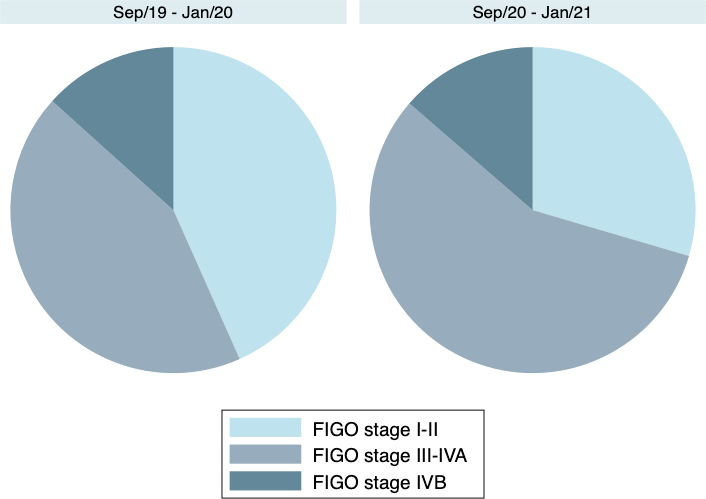
Cervical cancer stages during (September/20–January/21) and prior to (September/19–January/20) the COVID-19 pandemic, according to the 2018 FIGO Staging System.

Regarding cervical cancer, the proportion of patients with more advanced disease stages was also numerically higher in September/20–January/21, although the difference was not statistically significant (*p* = 0.328). Locally advanced disease (FIGO stages III–IVA) occurred in 56.8% (*N* = 25/44) of the cervical cancer patients in September/20–January/21 compared to 43.3% (*N* = 26/60) in September/19–January/20. Table 2 and Figure 2 show the distribution of 2018 FIGO stages during the two periods.

No difference was observed in terms of reasons (screening or symptomatic presentation) that lead to cervical cancer diagnosis during the two periods. Unfortunately, most patients were diagnosed due to symptomatic presentation rather than due to screening in both periods, with 92.9% diagnosed due to symptoms in September/20–January/21 and 91.7% in September/19–Jan/20 (*p* = 0.826).

### Intervals from biopsy to the first cancer centre visit

The intervals from tumour biopsy until the first cancer centre visit were not normally distributed (Shapiro–Wilk test < 0.001). Thus, data were compared using the Mann–Whitney test.

Median time from breast cancer tumour biopsy to first cancer centre visit was 5.4 months in September/20–January/21 and 6.7 months in September/19–January/20 (*p* Mann–Whitney < 0.001). Results were similar for cervical cancer, with a median time from tumour biopsy to first cancer centre visit of 4 months in September/20–January/21 and 6.1 months in September/19–January/20 (*p* Mann–Whitney = 0.010).

These results show that access to this particular cancer centre after the cancer diagnosis was not delayed. Actually, the time for the first cancer centre visit was lower during the pandemic than previous to it.

## Discussion

Our results showed that patients had more advanced-stage breast and cervical cancers at diagnosis during the pandemic compared to a similar period previous to the pandemic. The proportion of stage III breast cancer increased by 14.1%, and the proportion of stages III–IVA cervical cancer increased by 13.5%, although the difference was only statistically significant for breast cancer. The total number of new breast and cervical cancer cases in the tertiary cancer centre was also lower during the pandemic period. Another interesting finding was that the proportion of breast cancer cases diagnosed due to screening tests decreased during the pandemic.

On the other hand, after the cancer diagnosis was made, the interval to first cancer centre visit decreased during the pandemic compared to the previous period. Considering this, the main negative impacts of the COVID-19 pandemic for delaying cancer patients’ treatment seemed to be the decrease in screening and diagnostic procedures. Hawrot *et al* [8] also found in a retrospective cohort that time to treatment initiation after breast cancer diagnosis was maintained during the pandemic in the United States. The main hypothesis to explain the decrease of the interval to the first cancer centre visit observed in our centre is that fewer patients were referred during the pandemic, facilitating the access of those patients who were referred. Nevertheless, our cancer centre is a tertiary cancer centre focused only on oncologic patients. Less specialised centres, who also treat non-oncologic patients, might have required greater adjustments of health care focus to attend COVID-19 patients. In this scenario, we cannot rule out the possibility of delays in beginning cancer treatment after diagnosis.

In accordance with our finding, a previous study showed that breast cancer screening mammograms had an average decrease of 61.7% and a maximum decline of 94.6% during the pandemic in the United States [9]. In Brazil, in the city of Fortaleza, the decrease of screening mammograms was up to 95% [10]. In a cross-sectional study of the Brazilian public health system, the decline in mammograms reached 42% all over the nation from 2019 to 2020 [11]. In the case of cervical cancer, a Californian study showed that during the pandemic, when the population was recommended to stay at home, cervical cancer screening rates decreased by 78% [12].

The main concern of this decrease in screening tests is the worsening of cancer stages at diagnosis, as observed in our study, which jeopardises oncologic outcomes and prognosis. In addition, missing the screening tests leads to a loss of the opportunity to treat pre-malignant lesions. Indeed, in an estimate based on the British health system, interrupting the screening for cervical cancer for 6 months would result in an excess of about 630 cases of cervi cal cancer (4.3 per 100,000 women) [13]. A British modelling study showed that a 12-month delay in breast cancer diagnoses caused by the pandemic increases death rates by 7.9%–9.6% after 5 years [14]. Similarly, a Canadian mathematical model suggested that a 6-month would result in 670 extra advanced cancer cases and 250 additional deaths [15].

Despite these estimates, objective evidence confirming the negative impact of the COVID-19 pandemic on cancer cases was lacking. Corroborating our findings, two recent Italian retrospective cohorts showed a worsening of breast cancer stage at diagnosis due to COVID-19 [16, 17]. Toss *et al* [16] evaluated 400 patients and showed that a 2-month interruption in mammographic screening increased stage III breast cancer by 10.3% and node-positive disease by 11.2%. Vanni *et al* [17] confirmed an increase in tumour dimensions and N-staging in the COVID-19 era group compared to the pre-COVID-19 era. To the best of authors’ knowledge, the present study is the first to evaluate the impact of the COVID-19 pandemic on breast and cervical cancer stages based on objective real-world data in Latin America.

## Conclusion

At the first cancer centre visit, breast and cervical cancer stages were more advanced during the COVID-19 pandemic than previous to it. The proportion of breast cancer diagnoses due to screening tests also decreased during the pandemic. These results confirm the long-term negative impacts of the COVID-19 pandemic for oncologic patients. Thus, efforts should always be made not to compromise essential cancer services, such as screening tests, diagnostic procedures and curative treatments.

## Conflicts of interest

RCB has received grant and financial support for educational programmes from AstraZeneca; grant from Novartis; financial support for attending symposia from Roche and AstraZeneca; and personal fee for expert testimony from Ache, outside the submitted work. LT has received grant from Novartis, personal fee for expert testimony from MSD, Lilly, Novartis and Genomic Health, fee for non-continuing medical education services from Lilly, Novartis, Pfizer, Roche and Libbs, travel support from Pfizer, Roche, Libbs and United Medical, outside the submitted work. All other authors have no disclosure/conflict of interest.

## Funding statement

This research received no funding.

## Figures and Tables

**Table 1.** Breast cancer stages during (September/20–January/21) and prior to (September/19–January/20) the COVID-19 pandemic, according to the AJCC/UICC 8th edition TNM anatomic Staging System.

Period	Stage I *N* (%)	Stage II *N* (%)	Stage III *N* (%)	Stage IV *N* (%)	*p*-value^a^
September/19–January/20 (*N* = 457)	94 (29.6%)	198 (43.3%)	106 (23.2%)	59 (12.9%)	<0.001
September/20–January/21 (*N* = 268)	25 (9.3%)	101 (37.7%)	100 (37.3%)	42 (15.7%)	

a Chi-squared test

**Table 2.** Cervical cancer stages during (September/20–January/21) and prior to (September/19–January/20) the COVID-19 pandemic, according to the 2018 FIGO Staging System.

Period	Stage I *N* (%)	Stage II *N* (%)	Stage III *N* (%)	Stage IVA *N* (%)	Stage IVB *N* (%)	*p*-value^a^
September/19–January/20 (*N* = 60)	12 (20.0%)	14 (23.3%)	16 (26.7%)	10 (16.7%)	8 (13.3%)	0.328
September/20–January/21 (*N* = 44)	6 (13.6%)	7 (15.9%)	12 (27.3%)	13 (29.5%)	6 (13.6%)	

a Chi-squared test
